# Mesoporous One-Component Gold Microshells as 3D SERS Substrates

**DOI:** 10.3390/bios11100380

**Published:** 2021-10-09

**Authors:** Anna S. Vikulina, Inna Y. Stetsyura, M. Serdar Onses, Erkan Yilmaz, Andre G. Skirtach, Dmitry Volodkin

**Affiliations:** 1 Department of Theory and Bio-Systems, Max Planck Institute of Colloids and Interfaces, Am Mühlenberg 1, 14476 Potsdam, Germany; 2 Bavarian Polymer Institute, Friedrich-Alexander University Erlangen-Nürnberg (FAU), Dr.-Mack-Straße 77, 90762 Fürth, Germany; 3 Fraunhofer Institute for Cell Therapy and Immunology, Branch Bioanalytics and Bioprocesses, Am Mühlen-berg 13, 14476 Potsdam, Germany; inna_st@mail.ru; 4 ERNAM-Erciyes University Nanotechnology Application and Research Center, Kayseri 38039, Turkey; onses@erciyes.edu.tr (M.S.O.); erkanyilmaz@erciyes.edu.tr (E.Y.); 5 Department of Materials Science and Engineering, Faculty of Engineering, Erciyes University, Kayseri 38039, Turkey; 6 Department of Analytical Chemistry, Faculty of Pharmacy, Erciyes Üniversity, Kayseri 38039, Turkey; 7 Technology Research & Application Center (TAUM), Erciyes University, Kayseri 38039, Turkey; 8 Department of Biotechnology, Ghent University, Coupure Links 653, 9000 Ghent, Belgium; andre.skirtach@ugent.be; 9 School of Science and Technology, Nottingham Trent University, Clifton Lane, Nottingham NG11 8NS, UK; dmitry.volodkin@ntu.ac.uk

**Keywords:** calcium carbonate, vaterite, hard templating, Raman spectroscopy, microparticles

## Abstract

Surface-enhanced Raman scattering (SERS) is a powerful analytical tool for label-free analysis that has found a broad spectrum of applications in material, chemical, and biomedical sciences. In recent years, a great interest has been witnessed in the rational design of SERS substrates to amplify Raman signals and optionally allow for the selective detection of analytes, which is especially essential and challenging for biomedical applications. In this study, hard templating of noble metals is proposed as a novel approach for the design of one-component tailor-made SERS platforms. Porous Au microparticles were fabricated via dual ex situ adsorption of Au nanoparticles and in situ reduction of HAuCl_4_ on mesoporous sacrificial microcrystals of vaterite CaCO_3_. Elimination of the microcrystals at mild conditions resulted in the formation of stable mesoporous one-component Au microshells. SERS performance of the microshells at very low 0.4 µW laser power was probed using rhodamine B and bovine serum albumin showing enhancement factors of 2 × 10^8^ and 8 × 10^8^, respectively. The proposed strategy opens broad avenues for the design and scalable fabrication of one-component porous metal particles that can serve as superior SERS platforms possessing both excellent plasmonic properties and the possibility of selective inclusion of analyte molecules and/or SERS nanotags for highly specific SERS analysis.

## 1. Introduction

In the last decade, surface-enhanced Raman spectroscopy (SERS) has attracted increasing attention as a strong bioanalytical tool that possesses cost-effective, highly selective, and non-destructive multimodal testing [1]. Due to its versatility, SERS has provided solutions for diverse medical applications, including practical multiplex in vitro diagnostics [2] and tumor screening [3]. Besides this, the prominent multiplexing capabilities of Raman spectroscopy provide this technique with advanced capabilities for serving as an all-in-one single platform that combines in vivo diagnostics and therapy [4]. This theranostic strategy is a game changer for modern medicine allowing wide opportunities for personalized medicine. In this context, SERS has provided new modalities for label-free imaging that primarily find applications in clinical oncology [3,5].

The electromagnetic enhancement in SERS is commonly achieved via the effect of a plasmon resonance induced around metal nanoparticles (NPs) (typically silver or gold) and/or in the nanostructured materials serving as the SERS substrates [6]. The enhancement factor (EF) directly depends on the structure and plasmonic hot spots of SERS substrates, which makes their rational design and reproducible fabrication a key challenge in advancing SERS applications, which is also applicable for live cell imaging [7]. Research on SERS is ongoing [8] and colloidal NPs render several advantages, such as highly reproducible and cost-effective synthesis via bottom-up strategies and tailoring their optical properties via tuning particle shape and size, aggregation, surface functionalization, and the application of external stimuli (e.g., visible light irradiation [9]).

Plasmonic NPs made of noble metals are extensively employed as SERS platforms due to their ability to efficiently scatter and absorb light, allowing even single molecule detection [10]. Fabrication of metal NPs suitable for SERS applications is technically simple and cost effective. However, it is challenging to produce (particularly reproducibly) metal NPs-based SERS due to an uncontrollable colloid aggregation that might be accompanied by the loss of optical stability. Moreover, the size of single NPs seriously limits their applicability for cell imaging because these NPs are invisible by conventional optical microscopy; they are too small to be manipulated, and it is difficult to place them on the point of interest and to ensure their presence [11]. This drawback can be overcome by the self-assembly of colloidal NPs into homogeneous and ordered 2D micro-patterned layers and arrays, [1] or by fabrication of 3D SERS platforms which are easier to manipulate, generally have higher colloidal stability, and facilitate hot spot formation in all three dimensions. However, the fabrication of 3D SERS (micro)platforms is mostly expensive and still technologically challenging [12]. NP assembly is often very unpredictable, and the assembled particles are not structurally stable, which is especially the case for 3D assemblies [13].

In recent years, several approaches were proposed to solve these problems. The first strategy is based on the coating of various microparticles served as the cores (mainly polystyrene [11,14,15] and silica [16,17]) with metal NPs (Au [11,14], Ag [15,16,17]). Later, (meso)porous cores have also been applied demonstrating advantages in the formation of hot spots due to an increase in the density of electromagnetic fields [13,18,19]. Silica and carbonate particles are most frequently utilized as porous cores for NPs immobilization; one of the advantages of converting such particles into SERS sensors is their mobility [20]. Most of the reported 3D SERS platforms made on these porous matrices partially or in full retain the material of the matrix after fabrication. In most cases, the porous matrix was not eliminated, e.g., Ag/CaCO_3_ hybrids [18], Ag/Astralen/CaCO_3_ [21], Ag/Astralen/SiO_2_, and Au/Astralen/SiO_2_ [22]. In the study in [13], the polymer matrix was eliminated but silification that accompanied this process allowed for the fixing of the nanostructures while generating ‘negatively replicated’ porous structures. Complete elimination of the template leads to the disintegration of assembled plasmonic NPs back to singe NPs and their polydispersed aggregates [23]. Therefore, in all of these cases, the obtained microparticles were hybrid two- or multi-component 3D SERS platforms.

In contrast, here we introduce a new approach for the fabrication of one-component spherical porous SERS platforms. This is achieved via hard templating followed by complete elimination of the template, while the structure of SERS microparticles is preserved after the template elimination. Mesoporous vaterite CaCO_3_ microcrystals were used as sacrificial templates for the assembly of Au SERS platforms. Vaterite CaCO_3_ crystals attracted significant interest as a low-cost natural material that possesses no toxicity and allows for the encapsulation of macromolecules at mild conditions closed to physiological ones [24]. As a template, vaterite can be eliminated by the addition of chelating agents or slight acidification of the media (pH below neutral) [25]. Importantly, control over vaterite crystal size [26], shape [27], and porosity [28], without any additives, opens broad avenues for the utilization of these crystals as decomposable templates.

## 2. Materials and Methods

### 2.1. Materials

Ethylenediaminetetraacetic acid disodium salt dehydrate was purchased from AppliChem (Darmstadt, Germany). Tetrachloroauric (III) acid was purchased from ROTH (Karlsruhe, Germany). Potassium carbonate and rhodamine B were purchased from Fluka (Buchs, Germany). Calcium chloride dihydrate CaCl_2_·2H_2_O, sodium carbonate Na_2_CO_3_ anhydrous, ascorbic acid, dimethylaminopyridine (DMAP), and bovine serum albumin (Fraction V) were purchased from Sigma (Hamburg, Germany). Other reagents were from Sigma and were at least of analytical grade. Sterile deionized ultrafiltred water was used from a Millipore purification system (Darmstadt, Germany).

### 2.2. Synthesis of CaCO_3_ Templates

CaCO_3_ vaterite microcrystals were used as the templates for gold particles. The crystals were obtained by a precipitation reaction as described in [28], with slight modifications. Aqueous solutions of calcium chloride (1 M, 3 mL), sodium carbonate (1 M, 3 mL), and deionized water (9 mL) were simultaneously mixed at 22 °C. The agitation was carried out for 45 s under constant stirring on a magnetic stirrer at 160 rpm and 22 °C, followed by incubation during 60 min. Precipitated CaCO_3_ microcrystals were thoroughly washed with deionized water and dried at 80 °C. Reaction yield was above 95%.

### 2.3. Synthesis of Gold Nanoparticles

Au NPs (~0.6 mg mL^−1^) were produced via the classical Brust two-phase method in toluene without modifications and transferred into water using a phase transfer agent (DMAP).

### 2.4. Immobilization of Gold Nanoparticles

Immobilization of Au NPs on CaCO_3_ microcrystals was performed using one of three alternative methods: 1. Adsorption of ex situ synthesized Au NPs (0.5 mL, ~0.06 mg mL^−1^) into the pores of vaterite microcrystals dispersed in water was performed under continuous shaking for 30 min, followed by centrifugation and washing in deionized water. 2. In situ reduction of a tetrachloroauric acid. A solution of HAuCl_4_ (1.5 mL, 1%) was added to aqueous solution of K_2_CO_3_ (25 mg, 100 mL), and the mixture was shaken for 30 min. Then, a dispersion of vaterite microcrystals in water (0.2 mL) was mixed with the solution of HAuCl_4_ (4 mL) followed by the addition of L-ascorbic acid (10 µL, 1%). A reduction reaction was carried out until the solution changed color from colorless to blue and then violet, indicating the growth of Au NPs. Then, the reaction mixture was centrifuged followed by decantation and washing in deionized water several times. 3. The third method was a combination of the two previous methods. In the first step, adsorption was carried out similarly to the first method. After washing and removing the supernatant, a reaction of the HAuCl_4_ reduction technique was carried out as described above.

### 2.5. Fabrication of One-Component SERS Substrates

Gold porous microparticles were made by the dissolution of CaCO_3_ core via drop-by-drop addition of EDTA-Na_2_ aqueous solution (0.05 M) to an equal volume of obtained suspension, followed by centrifugation and washing in deionized water. The suspension was stored at 4 °C.

### 2.6. Dynamic Light Scattering (DLS) and ζ-Potential Measurements

Suspension of Au NPs in PBS was used for the measurements using Zeta-sizer Nano ZS (Malvern, UK).

### 2.7. Brunauer, Emmett and Teller (BET) Analysis

Nitrogen adsorption–desorption analysis of pure vaterite crystals was carried out using a QUADRASORB SI-MP (Quantachrome Instruments, Boynton Beach, FL, USA), 77.3 K. The samples were degassed at 150 °C for 20 h prior to the measurements. Porosity analysis was performed using the Barret–Joyner–Halenda model.

### 2.8. Scanning Electron Microscopy (SEM) and Transmission Electron Microscopy (TEM)

SEM images were recorded using a Carl Zeiss LEO 1550 electron microscope (Carl Zeiss, Jena, Germany) at an accelerating voltage of 30 kV. TEM images were recorded by a Carl Zeiss EM 912 electron microscope (Carl Zeiss, Jena, Germany) at an accelerating voltage of 20 kV. For TEM, the samples were prepared by microtome method.

### 2.9. Raman Microscopy

A Raman confocal microscope equipped with a piezo scanner (P-500, Physik Instrumente, Karlsruhe, Germany) and a diode-pumped 785 nm NIR laser excitation (Toptica Photonics AG, Graefelfing, Germany) was used. The laser beam was focused through a 60× water immersion (Nikon, NA = 1.0) or 100× (Olympus, NA = 0.95) microscope objective. The spectra were acquired with a thermoelectrically cooled CCD detector (DU401ABV, Andor, UK) behind grating (300 g mm^−1^) spectrograph (Acton, Princeton Instruments Inc., Trenton, NJ, USA) with a spectral resolution of 6 cm^−1^.

## 3. Results

**Figure 1:**
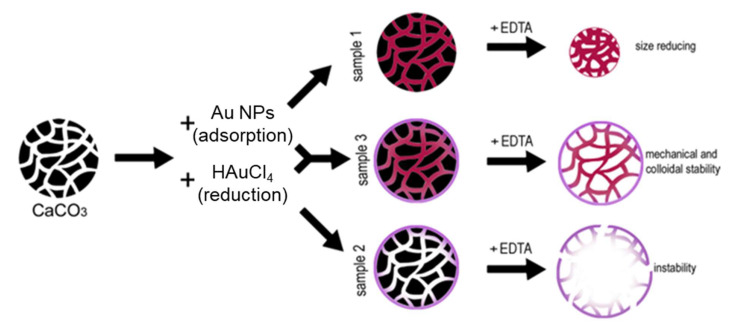
Three methods of fabrication of porous Au microparticles: sample 1, ex situ adsorption of Au NPs; sample 2, in situ reduction of HAuCl_4_; sample 3, adsorption of Au NPs followed by the reduction of HAuCl_4_ (combination of methods 1 and 2).

Spherical microcrystals of vaterite with an average diameter of 11 ± 1 µm were prepared in a pure aqueous solution by a mixing technique [28]. The crystals’ pore sizes in the range of 5–30 nm were confirmed by BET. Three methods of Au immobilization into the crystals were tested (Figure 1): (i) the immobilization of ex situ synthesized Au NPs into the crystals by means of adsorption; (ii) the in situ growth of Au NPs through reduction of AuHCl_4_; and (iii) the combination of the first and second methods, i.e., in situ Au NPs were synthetized in crystals with pre-adsorbed spherical Au NPs. Ex situ synthesized Au NPs had a spherical shape and were 19 ± 9 nm in diameter; their ζ-potential was found to be +16 ± 7 mV (Figure S1 in supplementary materials). These three methods resulted in the formation of unstable samples 1 and 2 and stable sample 3, respectively (Figure 1).

The carbonate template removal was induced by the addition of aqueous solution of EDTA (Figure S2 in supplementary materials). During the template elimination, sample 1 collapsed and significantly decreased in size with a shrinkage coefficient of 1.9 ± 0.2. Sample 2 was also unstable and disintegrated just after formation. Sample 3 was the only stable sample, which was fabricated by the combined method. After the dissolution of the cores, sample 3 particles underwent a negligible shrinkage that was below 5%.

**Figure 2:**
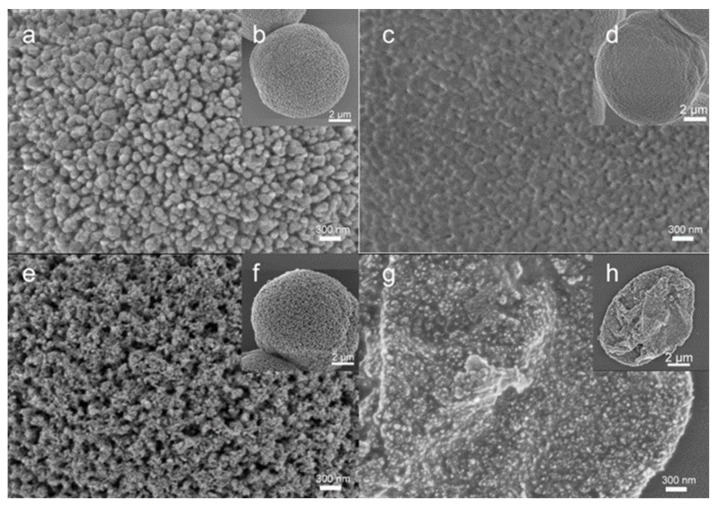
SEM images of: (**a**,**b**) CaCO_3_ microcrystals; (**c**,**d**) –Au NPs/CaCO_3_hybridsobtained by Au NP adsorption (method 1); (**e**,**f**) Au NPs/CaCO_3_ hybrids obtained by Au NP adsorption and further reduction of HAuCl_4_ (method 3); (**g**,**h**) porous Au microshells obtained by method 3 followed by CaCO_3_ template elimination with EDTA.

The structure of Au NPs/CaCO_3_ hybrids and one-component Au microparticles obtained after the CaCO_3_ elimination was investigated by means of SEM. Typical images of bare CaCO_3_ microcrystals and their external surface are shown in Figure 2a,b. The crystals have a highly developed mesoporous structure composed of nanocrystallines aggregated together during the formation of the crystal. Adsorption of Au NPs on the surface of the CaCO_3_ crystals changes the morphology of the initial crystal surface (Figure 2c,d). Smoothing of the crystals surface is evidence of the adsorption of Au NPs and their retention after multiple washing steps. Taking into account similarly low positive ζ-potentials of both vaterite (typically varies in the range from +11 to +15 mV [29]) and Au NPs, (+16 ± 7 mV), it is likely that NP adsorption is driven by non-electrostatic interactions. In fact, this is not unusual for the adsorption of different species on the vaterite. For instance, the significant role of hydrophobic interactions has been demonstrated for the adsorption of mucin on CaCO_3_ microcrystals [30]; another work demonstrated the dominating impact of van der Waals interactions for the adsorption of catalase [31]. Although the internal structure of hybrid CaCO_3_ crystals with Au NPs adsorbed on them remains invisible by SEM, one can also reasonably speculate about a partial adsorption of Au NPs on the internal surface of the crystal pores that have a diameter of 5–30 nm and therefore are largely accessible for the diffusion of 19 ± 9 nm NPs into the pores. However, it seems that the adsorption on the crystals’ external surface dominates over the adsorption into the pores; therefore, the final structure is not robust enough and undergoes shrinkage after the elimination of the template with EDTA (Figure S2). Furthermore, in this study we focused on the Au microshells successfully fabricated via NP adsorption followed by HAuCl_4_ reduction. Au microshells were stored in an aqueous suspension and kept colloidal stability and morphology at least for a few weeks.

The formation of an Au coating by NP adsorption followed by HAuCl_4_ reduction leads to the formation of a dense coating with a highly developed morphology. SEM images (Figure 2e,f) show the assembly of Au NPs forming a nanostructured coating on the templates, wherein larger pores most likely correspond to the initial pores of vaterite crystals. This Au coating is robust enough to keep the integrity of the Au microparticles formed after the dissolution of the CaCO_3_ template (Figure 2g,f). Previous studies already demonstrated complete elimination of the template by the addition of EDTA, even in the presence of polymer multilayers [32] and TiO_2_ NPs [33], which, together with no steric limitations expected for the release of the products of CaCO_3_ dissolution (free ions and Ca-EDTA complex) from the mesopores, suggests the fabrication of one-component Au microparticles. These Au microshells maintain a porous structure on their surface representing a kind of thick porous shell that collapsed (spherical shape lost but integrity kept) during the sample drying as a required step for SEM imaging.

**Figure 3:**
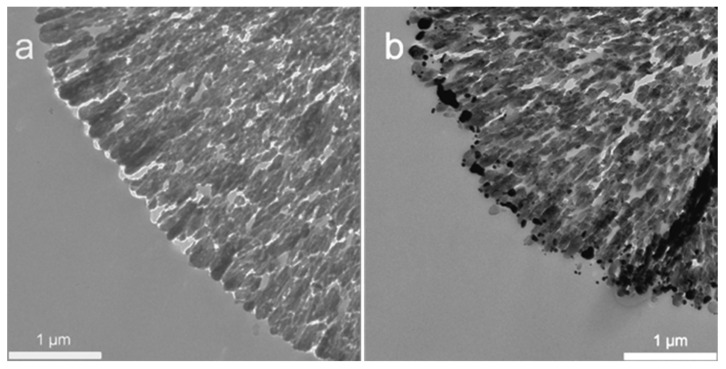
TEM images of: (**a**) CaCO_3_ microcrystal; (**b**) Au NPs/CaCO_3_ hybrids obtained by Au NP adsorption followed by the reduction of HAuCl_4_ (method 3) before the elimination of CaCO_3_.

Since SEM imaging does not provide information on the internal structure of the hybrids, additional characterization by TEM was performed (Figure 3). Bare CaCO_3_ crystals (Figure 3a) demonstrate a typical dendritic microstructure comprising sub-particles (the aggregated nanocrystallines) interconnected with each other forming the pores of tens of nm or more. The Au coating via the dual “adsorption–reduction” approach (method 3) leads to the formation of a dense Au shell composed of spherical nano-sized aggregates of Au (Figure 3b). It is known that non-linear intra- and inter-band transitions driven by aggregation effect promote changes in the sensitivity of the system [34]. Such aggregation behavior is well known for the metal NPs [18,25]. The average size of Au aggregates was 100 ± 62 nm as determined from TEM images. Notably, Au NPs also partially penetrate into the CaCO_3_ crystals. Although there is no complete filling of the pores with Au NPs, it ensures sufficient stabilization of the formed Au microshells after CaCO_3_ elimination as demonstrated by SEM (Figure 2d). To the best of our knowledge, this is the first successful attempt to fabricate one-component Au microparticles via the hard templating approach. In previous reports, composite vaterite–Au hybrid structures were used as the SERS platforms either without CaCO_3_ elimination (i.e., as composite substrates [18]) or following the elimination of CaCO_3_ that caused complete disintegration of metal coating back into the NPs or their small aggregates [23]. We assume that the dual “adsorption–reduction” formation of metal coating leads to more robust and stable nanostructures that endow the whole microparticle with significant stability after the template dissolution. Understanding of the impact of two individual processes, adsorption and reduction, on the thickness and stabilization of the Au coating is an interesting question; however, it was out of the scope of this study. Therefore, inspired by the interconnectivity of nano-structured Au clusters that may improve metal plasmonic properties, we further evaluated the performance of porous Au microshells as SERS platforms.

**Figure 4:**
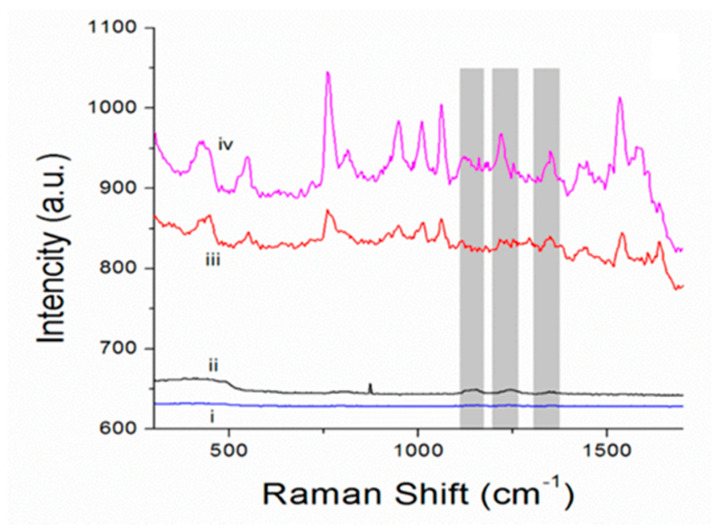
Raman spectra of dried BSA: (**i**) at 0.4 µW and (**ii**) at 40 mW laser power, respectively. (**iii**) Raman spectrum of a water dispersion of porous Au microshells; (**iv**) SERS spectrum of BSA (2 mg/mL) in the presence of porous Au microshell dispersion, laser power 0.4 µW. The characteristic scattering lines of BSA are marked with grey color.

Bovine serum albumin (BSA) was used as a probe to evaluate the SERS performance of the one-component Au microshells at characteristic modes of albumin (~1130 cm^−1^, ~1180 cm^−1^, ~1210 cm^−1^, and ~1345 cm^−1^ [35]). The Raman spectrum of pure BSA was recorded at different laser powers, starting from 0.4 µW until the characteristic modes of BSA were recorded with sufficient resolution (40 mW); these boundary spectra are shown in Figure 4. The Raman spectra of the obtained one-component porous Au microshells in water dispersion have typical modes for the DMAP used for the stabilization of Au NPs [36]). The same peaks are observed in SERS spectrum of BSA obtained in its dispersion with porous SERS platforms. Corresponding characteristic peaks of the analyte are shown in SERS spectrum with the grey lines (Figure 4). SERS spectra were recorded at minimum 0.4 µW laser power and provide solid evidence of a very high sensitivity of the obtained pure porous Au-based microshells as SERS platforms.

The surface enhancement factor (EF) was calculated as the concentration-corrected and laser intensity-corrected ratio (Equation (1)) [37]:

(1) $$EF=\frac{I_{SERS}}{I}\cdot \frac{n}{n_{SERS}}\cdot \frac{I_{laserSERS}}{I_{laser}}$$

where *I_SERS_*—peak intensity at SERS signal; *I*—peak intensity at conventional Raman signal; *n*—the concentration of the analyte at recording of the conventional Raman signal; *n_SERS_*—the concentration of the analyte at recording of SERS signal; *I_laser_*—laser intensity at recording of conventional Raman signal; *I_laser SERS_*—laser intensity at recording of SERS signal.

An EF of 7.6 ± 1.6 × 10^8^ was achieved for BSA. In addition, we verified the analytical performance of porous Au microshells for detection of Rhodamine B (Figure S3 in supplementary materials). For this case, Au NPs stabilized with cetrimonium bromide (CTAB) were utilized. An EF of 2 × 10^8^ was achieved for Rhodamine B. These EFs are about three to five orders of magnitude higher than those reported in the literature for the determination of rhodamine 6G and TRITC-BSA using polycaprolactone-based scaffolds modified with vaterite CaCO_3_ and Ag NPs as the SERS substrate [37], as well as those reported for the determination of the small dyes using soft templated porous gold–silica hybrid microspheres [13] and hybrid CaCO_3_/Ag microparticles used for SERS without CaCO_3_ elimination [18]. Although it should be noted that the approaches used for the calculations of the EFs in two latter cases differed from Equation (1), which is an obstacle to direct comparison of these results, such high EFs demonstrate high potential of the fabricated one-component SERS platforms. At the same time, it should be noted that there are some hurdles in the understanding and interpretation of the SERS signal coming from the background (Figure 4iii). While the peaks in the region of 700–1100 cm^−1^ are clearly attributed to the DMAP used to stabilize NPs, rather high and non-uniform overall background Raman intensity generated by Au microshells might seriously limit single analyte detection and multiplexing in the complex media and real samples. This represents a current drawback of the microshells designed in this study; therefore, further optimization and a proper evaluation of their analytical performance is required in the future. Figure 3 suggests a dense interconnected architecture of Au microshells rather than formation of a homogeneous monolayer. This issue can be addressed, in particular, by control of localization of the NPs on the surface of the template which likely plays the pivotal role in plasmonic response modulation [38]. In this context, the interplay between reduction and adsorption approaches as well as control by template morphology (size, shape, and porosity) and conditions of microshell fabrication (ionic strength and temperature) can be investigated.

Moreover, the elevated background of Raman signal in the presence of the analyte observed for porous Au microshells deserves a separate discussion. Earlier, similar enhancement was reported for other SERS substrates, particularly those having structured nanovoid surfaces. This has been attributed to chemical metal–analyte interactions to the generation of the background signal making it dependent on the nature of both the metal and the molecule [39]. Applying this to Au microshells designed in this study, it seems that such background enhancement is pronounced due to their mesoporous structure that allows analyte adsorption.

## 4. Conclusions and Perspectives

In perspective, it should be noted that the control of the obtained pore sizes and internal structure in such SERS platforms can be achieved via the design of the vaterite microcrystals used as decomposable templates [28]. The structure of the microshells will be an inverted replica of the internal structure of the vaterite crystals. Together with mild vaterite decomposition conditions, this opportunity opens broader avenues for further utilization of such SERS platforms. Another important advantage of this platform is the ability to selectively locate probe macromolecules inside the microshell pores by pre-loading of the probes into the vaterite crystals; this is especially interesting, because mesopores of vaterite and those of Au microshells are in the same dimension as typical biomacromolecules [40]. Such a host–guest approach for SERS selectivity has several advantages in comparison, for example, with antibody–antigen binding-based techniques. This technology has an essential shortcoming; antibodies have Raman peaks that can bring superfluous noise in the Raman spectrum and reduce the usefulness of this method. Moreover, the porosity of the vaterite crystals opens the opportunity for inclusion of the nanotags for indirect SERS analysis, a concept that has gained popularity for selective determination of the biomarkers [41].

Finally, the dual NP “adsorption–reduction” approach proposed in this work represents a novel cost-effective and quick method for the fabrication of mesoporous SERS substrates. We believe that this study offers a novel strategy for the design of nanostructured 3D SERS substrates that can host tags to reach specific biomarker–tag interaction and achieve enhanced signals for specific interactions, which is the most challenging task in SERS.

## Supplementary Materials

The following are available online at https://www.mdpi.com/article/10.3390/bios11100380/s1. Figure S1: Zeta-potential distribution for the aqueous dispersion of Au NPs used in this study. Figure S2: Light transmittance images of the particles prepared by one of three methods (sorption, reduction, or sorption+reduction) before (0 s), during (30 s), and after complete dissolution of the CaCO_3_ (60 s); CaCO_3_ was dissolved by addition of EDTA-Na_2_ to the suspension of CaCO_3_/Au hybrid particles. Figure S3. Raman spectra of dried Rhodamine B: (i) at 0.4 µW and (ii) at 40 mW laser power, respectively. (iii) Raman spectrum of a water dispersion of porous Au microshells; (iv) SERS spectrum of Rhodamine B in the presence of porous Au microshell dispersion, laser power 0.4 µW. The characteristic modes of rhodamine B are marked with grey color. Au microshells were prepared by dual sorption+reduction using AuHCl_4_ and Au NPs stabilized by CTAB (5 nm, Sigma-Aldrich).
