# Supplementary material for: Mesoporous One-Component Gold Microshells as 3D SERS Substrates
Source: Biosensors (Basel). 2021 Oct 9;11(10):380. doi: 10.3390/bios11100380 (PMC8533941; doi:10.3390/bios11100380)
Supplement: Supplementary file 1 [file biosensors-11-00380-s001.zip › biosensors-1377723-supplementary.pdf]

biosensors

MDPI

Communication

Mesoporous One-Component Gold Microshells as 3D SERS Substrates

Anna S. Vikulina [1,2,*], Inna Y. Stetsyura [3], M. Serdar Onses [4,5], Erkan Yilmaz [4,6,7], Andre G. Skirtach [8] and Dmitry Volodkin [9]

[1] Department of Theory and Bio-Systems, Max Planck Institute of Colloids and Interfaces, Am Mühlenberg 1, 14476 Potsdam, Germany
[2] Friedrich-Alexander University Erlangen-Nürnberg (FAU), Bavarian Polymer Institute, Dr.-Mack-Straße 77, 90762 Fürth, Germany;
[3] Fraunhofer Institute for Cell Therapy and Immunology, Branch Bioanalytics and Bioprocesses, Am Mühlenberg 13, 14476 Potsdam-Golm, Germany; inna_st@mail.ru
[4] ERNAM-Erciyes University Nanotechnology Application and Research Center, Kayseri 38039, Turkey; onses@erciyes.edu.tr (M.S.O.); erkanyilmaz@erciyes.edu.tr (E.Y.)
[5] Department of Materials Science and Engineering, Faculty of Engineering, Erciyes University, Kayseri 38039, Turkey
[6] Department of Analytical Chemistry, Faculty of Pharmacy, Erciyes Üniversity, Kayseri 38039, Turkey
[7] Technology Research & Application Center (TAUM), Erciyes University, Kayseri 38039, Turkey
[8] Department of Biotechnology, Ghent University, Coupure Links 653, 9000 Ghent, Belgium; andre.skirtach@ugent.be
[9] School of Science and Technology, Nottingham Trent University, Clifton Lane, Nottingham NG11 8NS, UK; dmitry.volodkin@ntu.ac.uk
* Correspondence: anna.vikulina@fau.de

Citation: Vikulina, A.S.; Stetsyura, I.Y.; Onses, M.S.; Yilmaz, E.; Skirtach, A.G.; Volodkin, D. Mesoporous One-Component Gold Microshells as 3D SERS Substrates. *Biosensors* **2021**, *11*, 380. https://doi.org/10.3390/bios11100380

Received: 27 August 2021
Accepted: 3 October 2021
Published: 9 October 2021

Publisher's Note: MDPI stays neutral with regard to jurisdictional claims in published maps and institutional affiliations.

Copyright: © 2021 by the authors. Licensee MDPI, Basel, Switzerland. This article is an open access article distributed under the terms and conditions of the Creative Commons Attribution (CC BY) license (https://creativecommons.org/licenses/by/4.0/).

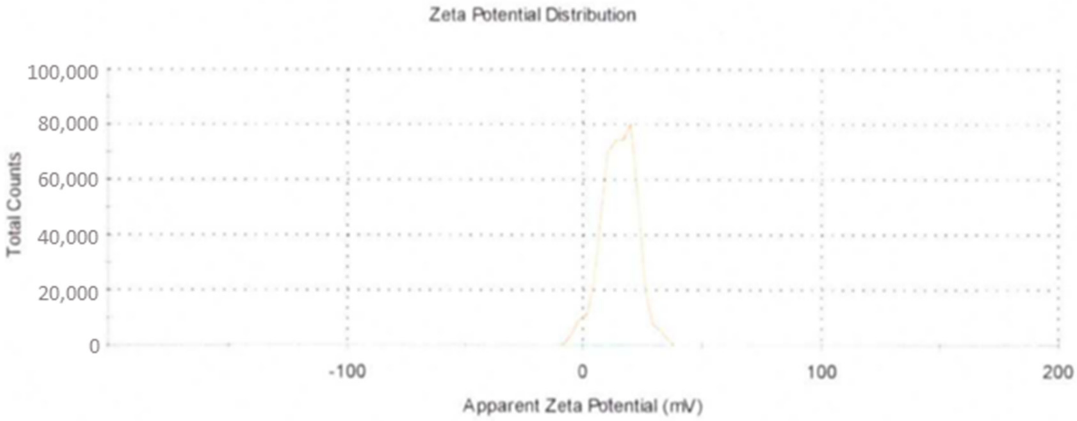

Figure S1. Zeta-potential distribution for the aqueous dispersion of Au NPs used in this study.

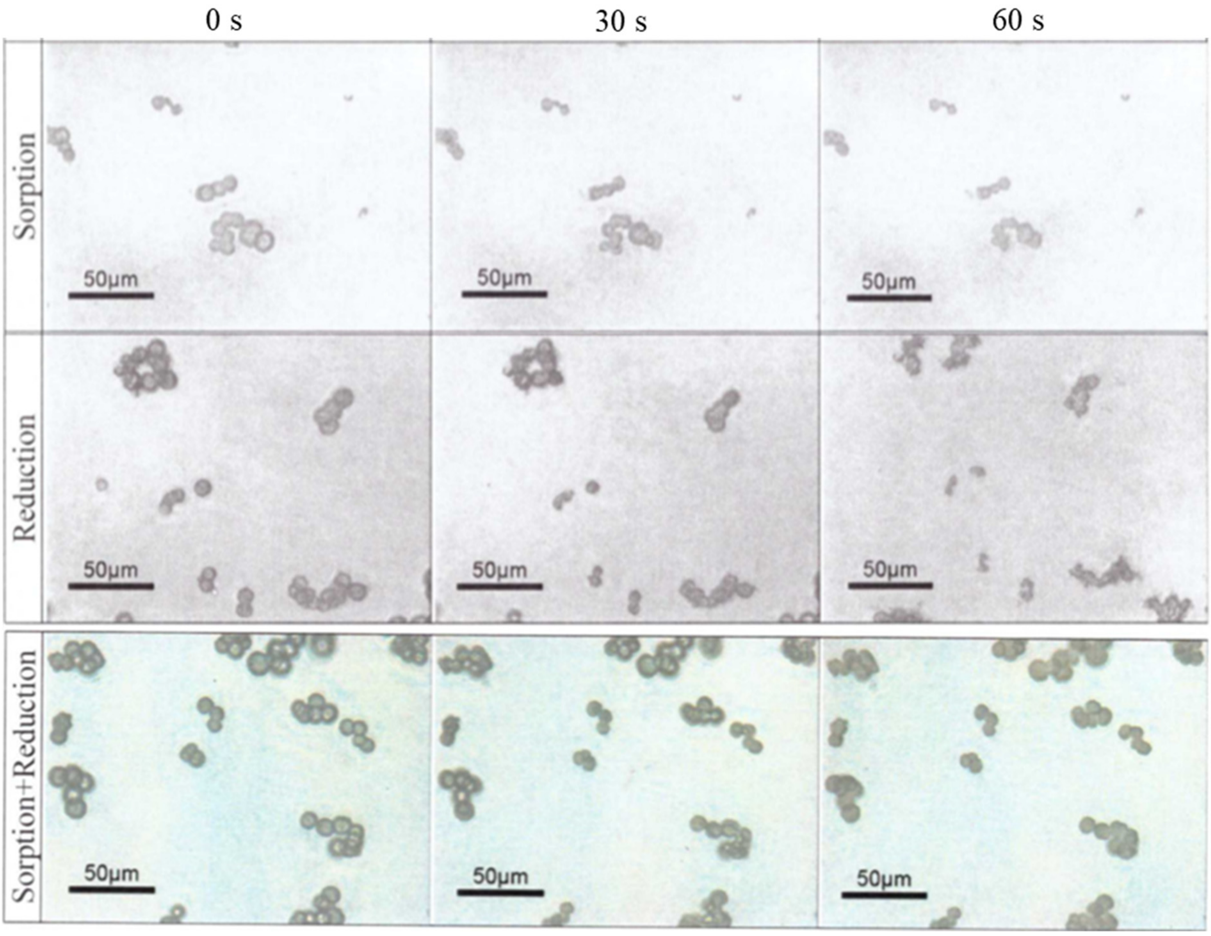

Figure S2. Light transmittance images of the particles prepared by one of three methods (sorption, reduction, or sorption+reduction) before (0 s), during (30 s), and after complete dissolution of the $CaCO_3$ (60 s); $CaCO_3$ was dissolved by addition of EDTA-Na_2 to the suspension of $CaCO_3$/Au hybrid particles.

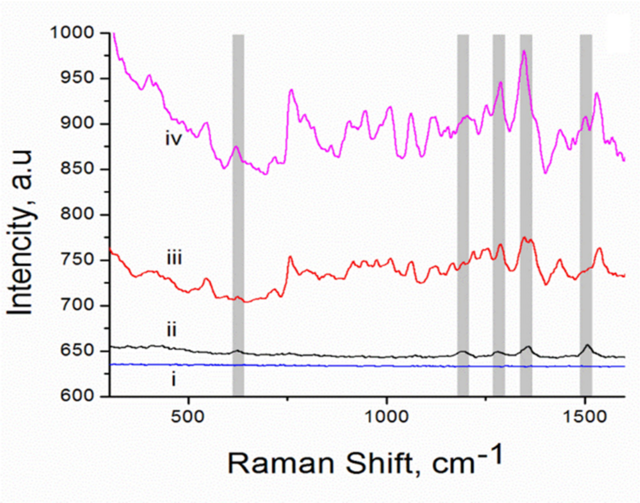

Figure S3. Raman spectra of dried Rhodamine B: (**i**) at 0.4 μW and (**ii**) at 40 mW laser power, respectively. (**iii**) Raman spectrum of a water dispersion of porous Au microshells; (**iv**) SERS spectrum of Rhodamine B in the presence of porous Au microshell dispersion, laser power 0.4 μW. The characteristic modes of rhodamine B are marked with grey color. Au microshells were prepared by dual sorption+reduction using $AuHCl_4$ and Au NPs stabilized by CTAB (5 nm, Sigma-Aldrich, Hamburg, Germany).
